# Circulating and Adipose Tissue Fatty Acid Composition in Black South African Women with Obesity: A Cross-Sectional Study

**DOI:** 10.3390/nu12061619

**Published:** 2020-05-31

**Authors:** Pamela A. Nono Nankam, Paul J. van Jaarsveld, Elin Chorell, Melony C. Fortuin-de Smidt, Kevin Adams, Matthias Blüher, Tommy Olsson, Amy E. Mendham, Julia H. Goedecke

**Affiliations:** 1 Division of Exercise Science and Sports Medicine, Department of Human Biology, University of Cape Town, Cape Town 7700, South Africa; mcfortuindesmidt@gmail.com (M.C.F.-d.S.); kevin@academyoplasticsurgery.co.za (K.A.); amymendham21@gmail.com (A.E.M.); julia.goedecke@uct.ac.za (J.H.G.); 2 Department of Endocrinology, Faculty of Medicine, University of Leipzig, 04103 Leipzig, Germany; Matthias.Blueher@medizin.uni-leipzig.de; 3 Non-Communicable Diseases Research Unit, South African Medical Research Council, Cape Town 7505, South Africa; Paul.vanJaarsveld@mrc.ac.za; 4 Division of Medical Physiology, Faculty of Medicine and Health Sciences, Stellenbosch University, Tygerberg 7505, South Africa; 5 Department of Public Health and Clinical Medicine, Umeå University, SE-907 42 Umeå, Sweden; elin.chorell@umu.se (E.C.); tommy.g.olsson@umu.se (T.O.); 6 Helmholtz Institute for Metabolic, Obesity and Vascular Research (HI-MAG) of the Helmholtz Zentrum München at the University of Leipzig and University Hospital Leipzig, 04103 Leipzig, Germany

**Keywords:** fatty acids metabolism, desaturase enzyme indices, erythrocytes, subcutaneous adipose tissue, body composition, insulin sensitivity

## Abstract

Background and Aims: During positive energy balance, excess lipid storage in subcutaneous adipose tissue (SAT) is associated with increased lipolysis. Elevated circulating fatty acid (FA) concentrations from both SAT lipolysis and dietary fat intake may result in visceral adipose tissue (VAT) accumulation, impairment of glucose metabolism, altogether increasing obesity-associated metabolic risks. We aimed to test the hypothesis that FA composition of red blood cell total phospholipids (RBC-TPL) and SAT is associated with body fat centralisation (VAT/SAT ratio) and insulin sensitivity (S_I_) in black South African women with obesity. Methods: Participants’ (*n* = 41) body fat composition and distribution, S_I_, and RBC-TPL, abdominal and gluteal SAT (gSAT) FA composition (gas-liquid chromatography) were measured. Results: RBC-TPL contained higher proportions of saturated fatty acids (SFAs) than SAT (*p* < 0.001), which were associated with lower S_I_ (*p* < 0.05). Mono-unsaturated fatty acids (MUFAs) and stearoyl-CoA desaturase-1 (SCD1)-16 were lower, while poly-unsaturated fatty acids (PUFAs), and delta-5 and delta-6 desaturase indices were higher in RBC-TPL than SAT (*p* < 0.001). Interestingly, FA profiles differed between SAT depots with higher SFAs and lower MUFAs, SCD1-16 and SCD1-18 indices in abdominal compared to gluteal SAT (*p* < 0.01). In both SAT depots, higher SFAs and lower PUFAs (n-3 and n-6) correlated with lower VAT/SAT ratio; and lower PUFAs (n-3 and n-6) and higher total MUFA correlated with higher S_I_. Conclusion: Our findings confirm the relationships between the FA composition of RBC-TPL and SAT and metabolic risk in black women with obesity, which are dependent on both the FA class, and the tissue type/blood compartment in which they are distributed.

## 1. Introduction

Obesity is associated with insulin resistance (IR) and this relationship is dependent on body fat distribution [1,2,3], which varies between ethnicities [4]. In South Africa (SA), the prevalence of obesity is higher in populations of African ancestry, especially in black women [5] who present with less visceral adipose tissue (VAT) and greater peripheral subcutaneous adipose tissue (SAT) than their white counterparts [4,6]. Paradoxically, despite this ‘favorable’ body fat phenotype, black African women are more insulin resistant than women of European descent [4,6]. The causes of these differences in insulin sensitivity are not yet fully understood and could be derived not only from differences in body fat distribution but also from variations in dietary fat intake [7,8]. Indeed, dietary fat intake differs between ethnicities with a lower intake of saturated fatty acid (SFA) and higher n-6 polyunsaturated fatty acid (PUFA) intake in black African women compared to their white counterparts [4,8]. This ethnic-specific dietary intake has been suggested to explain the dissimilarities in fatty acid (FA) metabolism and composition, basal insulin sensitivity and secretion and, consequently, ethnicity-specific variance in insulin sensitivity [9,10,11].

Long-term dietary fat intake is more accurately evaluated by the measurement of the FA composition of red blood cell total phospholipids (RBC-TPL), compared to plasma or serum FA profiles which only reflect the dietary FA intake of individuals for the last 2–3 weeks [12,13,14,15]. Alterations in RBC-TPL FA composition have been reported in insulin-resistant patients and were suggested to be derived either from modifications in dietary FA intake or changes in desaturase enzymes activities such as stearoyl-CoA desaturase-1 (SCD1), delta-5-desaturase (D5D) and delta-6 desaturase (D6D) [16]. Indeed, higher SCD1 and D6D and lower D5D activity have been associated with the impairment of insulin sensitivity in individuals with obesity [17,18,19]. Moreover, alterations in the composition of cell membrane phospholipids in insulin-sensitive tissues (e.g., AT, liver and muscle) may change membrane fluidity and consequently impair insulin signaling pathways [16]. 

Adipose tissue (AT) represents the major storage site of excess lipids in the form of triglycerides during positive energy balance, with an increase in adipocyte number and/or size resulting in the expansion of tissue mass [20]. The metabolic effects of AT expansion vary with its location and are dependent on the size of the depot. Indeed, excess lipid storage in SAT may cause the impairment of tissue metabolism and function [21]. Although VAT accumulation is considered to be highly linked to adverse metabolic effects [21], SAT accumulation can also contribute to the development of T2D, especially among black SA women, with several putative mechanisms identified [3]. Among others, increasing SAT basal and/or stimulated lipolysis during obesity elevates circulating free fatty acid (FFA) concentrations, which may interfere with glucose transport/uptake and phosphorylation activity, contributing to impaired peripheral insulin sensitivity [14,22]. Furthermore, these FFAs can be redirected to ectopic regions including VAT, thus, increasing obesity-associated metabolic risks [21]. VAT accumulation may therefore be influenced by SAT lipid metabolism and circulating FA profiles.

Consequently, it has been suggested that the AT depot-specific metabolic effects are partly exerted via differences in lipids and FA metabolism [21,23]. In this regard, the rate of FA synthesis, uptake and release, as well as FA mobilization and endogenous synthesis, differs between SAT depots (mainly abdominal and gluteal SAT) [24,25,26]. Furthermore, distinctive FA profiles of SAT depots have been reported [27]. However, there is a lack of recent studies comparing the FA composition between SAT depots and investigating their relationship with obesity-associated metabolic risks. We therefore aimed to test the hypothesis that the FA compositions of RBC-TPL and depot-specific SAT are associated with central body fat distribution and insulin sensitivity in black South African women with obesity. 

## 2. Methods

### 2.1. Study Participants and Ethical Considerations

Data in this cross-sectional study were baseline data collected as part of a 12-week aerobic-resistance training study, which will be published elsewhere. The detailed design of the study, including participant enrollment, inclusion criteria and screening procedures, has been previously published in the protocol paper [28]. Participants were recruited from universities and community groups in Cape Town, SA. They were included in the study if they were women aged between 20–35 years of isiXhosa ancestry (both parents) with stable body weight (i.e., <5% change from maximum weight for the last 6 months). Exclusion criteria were: (1) metabolic or inflammatory diseases (e.g., HIV, tuberculosis, active hepatitis, or rheumatoid arthritis), (2) smoking, (3) pregnancy, (4) lactation, (5) use of injectable contraception, which is the common contraception method in this population. Based on these criteria, a total of 41 black SA women with a BMI of 30–40 kg/m^2^ could be included in the present study. The study has been approved by the Human Research Ethics Committee of the Faculty of Health Sciences at the University of Cape Town (HREC REF: 827/2016). This study was registered to the Pan-African Clinical Trial Registry (trial registration: PACTR201711002789113) and was performed following the principles of the Declaration of Helsinki (1964, amended last in Fortaleza Brazil, 2013), the International Conference on Harmonization-Good Clinical Practice (ICH-GCP), and the laws of SA. All participants provided verbal and written informed consent before their involvement in any aspect of the study. 

In brief, anthropometry, body composition (including VAT and SAT areas) and fat distribution parameters were measured. Insulin sensitivity was measured using a frequently sampled intravenous glucose tolerance test (FSIGT) and SAT samples were collected for the analyses of FA composition. Participants’ usual dietary intake was estimated using a self-reported 7-day food frequency questionnaire, and energy and macronutrient intakes were analyzed as previously described [29]. 

### 2.2. Body Composition and Body Fat Distribution

As previously described [28], basic measures of anthropometry included weight, height, waist circumference (WC) and hip circumference (HC); whole-body composition was measured by dual-energy x-ray absorptiometry (DXA; Discovery-W, software version 12.7.3.7; Hologic, Bedford, MA, USA) according to standard procedures. Regional body fat distribution (gynoid and android fat as a proportion of total fat mass (%FM)) was characterized as previously described [30,31]. After a standardized meal (Energy: 2553 kJ), magnetic resonance imaging (MRI) was used to determine VAT and SAT volumes using a 3 Tesla whole-body human MRI scanner (MAGNETOM Skyra, Siemens Medical Solutions, Erlangen, Germany) as previously described [32]. 

### 2.3. Fasting Blood Samples and Frequently Sampled Intravenous Glucose Tolerance Test

Participants stayed overnight at the laboratory and were given a standardized evening meal at 8 PM before overnight fasting. The standardized meal was typical of their usual dinner intake, which contained 2456 kJ energy (E), 21 g protein (14% E), 49 g carbohydrate (33% E), and 32 g fat (48% E). At 7 AM, fasting blood samples were collected for the determination of RBC-TPL FA composition. After fasting blood collection, participants underwent an FSIGT. Baseline blood samples were collected (at −5 and −1 min) before the intravenous administration of glucose (50% dextrose; 11.4 g/m^2^ body surface area) over 60 s. After 20 min, human insulin (0.02 U/kg; NovoRapid, Novo Nordisk) was infused over 5 min (HK400 Hawkmed Syringe Pump, Shenzhen Hawk Medical Instrument Co., Shenzhen, China) and samples were subsequently collected for the determination of plasma glucose and serum insulin concentrations as previously described [28]. Insulin sensitivity (S_I_) was calculated using Bergman’s minimal model of glucose kinetics [33].

### 2.4. Adipose Tissue Sample Collection

Participants underwent fat biopsies (mini-liposuction) for SAT sample collection after 4–6 h of fasting for the subsequent determination of FA composition in abdominal SAT (aSAT; around the umbilicus) and gluteal SAT (gSAT; right upper outer quadrant) as previously described [28]. Approximately 2–3 cm^3^ of fat tissue was extracted from each site, washed with normal saline until no blood was visible, immediately frozen in liquid nitrogen (N_2_) and stored at −80°C until further analyses.

### 2.5. Determination of RBC-TPL and SAT Fatty Acid Composition

As previously described in [28], FA composition in RBC-TPL and SAT samples was evaluated using pairwise analyses that ensured samples from a participant were analyzed in the same batch on the same day. Aliquots of saline-washed RBCs (300 µL) and SAT (100 mg) were subjected to total lipid extraction with chloroform (amylene stabilised):methanol (2:1; vol:vol; containing 0.01% butylated hydroxytoluene) by using a modification of the method of Folch et al. [34,35]. Thin-layer chromatography (TLC) was applied to isolate the TPL fraction in the RBC total lipid extract [35]. The TLC-isolated RBC-TPL fractions and a small aliquot of the AT total lipid extracts were trans-methylated using methanol:sulphuric acid (95:5; vol:vol) at 70 °C for 2 h to yield fatty acid methyl esters (FAMEs) which, after cooling, were extracted with water and *n*-hexane. The organic layer containing the FAMEs was evaporated, redissolved in a small volume of *n*-hexane and analyzed by gas-liquid chromatography, as described in [35]. The sample FAMEs were identified by comparison of the retention times with those of a standard FAME mixture (27 FAMEs; Nu-Chek Prep Inc., MN, USA). The relative percentage of a FAME was calculated by taking the area under the curve (AUC) of a given FAME as a percentage of the total area count of all the FAMEs identified in the sample (%, wt:wt). Product-to-precursor FA ratios of the samples were used as a proxy indices to reflect estimated desaturase enzyme activities: 18:3n-6/18:2n-6 (gamma-linolenic acid; GLA/linoleic acid; LA) for D6D and 20:4n-6/20:3n-6 (arachidonic acid; AA/dihomo-gamma-linolenic acid; DGLA) for D5D activity [17,36]. SCD1 activity was estimated by the ratios of 16:1n-7/16:0 (for SCD1-16) and 18:1n-9/18:0 (for SCD1-18) [37]. This method has been well established as an approach to estimate the activity of desaturase enzymes in humans [17,38,39].

## 3. Statistical Analyses

Data were expressed as mean ± standard deviation (SD) or median interquartile range (25th–75th percentile) depending on the normality of quantitative variables. Normality was tested using the Shapiro–Wilk test and data were log-transformed before analysis if p values from the normality test were lower than 0.05. The comparison of FA composition between RBC, aSAT and gSAT was performed using a one-way repeated measures ANOVA (using Stata software v.13.1). The relationship between FA profiles in each tissue type and VAT/SAT ratio and S_I_ were explored using projection based multivariate analysis. Firstly, the data set was inspected by a principal component analysis (PCA) to detect groupings, trends and outliers. The associations between tissue-specific FA composition and VAT/SAT ratio and S_I_ were subsequently explored using orthogonal partial least squares of analyses (OPLS). All models were validated based on ANOVA of the cross-validated OPLS scores (CV-ANOVA) for significance testing [40]. All OPLS analyses were performed using SIMCA v.16. FAs were considered significant when fulfilling the statistical significance criteria using post-hoc linear regression on loadings calculated from the validated OPLS models on a 95% confidence level [41].

## 4. Results

### 4.1. Participant Characteristics and Dietary Intake

The participants involved in this cross-sectional study were young (23 (21–27) years), obese (33.9 ± 2.8) kg/m^2^) women (Table 1). The time course response of the glucose, insulin and c-peptide in response to the FSIGT have been recently published [32]. Self-reported dietary fat intake comprised of 34% of total energy (%E), of which 9.5%E was from SFA, 11.4%E from MUFA and 7.9%E from PUFA (Table 2).

### 4.2. Tissue-Specific Fatty Acid Composition

The FA composition of RBC-TPL was 44% SFA, 16% MUFA and 40% PUFA, while gSAT comprised of 31% SFA, 43% MUFA and 26% PUFA and aSAT comprised of 32% SFA, 42% MUFA and 26% PUFA (Table 3). Accordingly, total SFA and PUFA were higher, while total MUFA was lower in RBC-TPL compared to SAT depots (*p* < 0.001). When comparing these FA classes between SAT depots, total SFA was higher and total MUFA was lower in aSAT than in gSAT (*p* < 0.01), with no difference in total PUFA content (*p* > 0.05). 

When comparing individual FAs in RBC-TPL and SAT depots (Table 3), long-chain SFAs (18:0, 20:0, 22:0 and 24:0) were higher and the medium-chain SFA 14:0 was lower in RBC-TPL than in SAT depots (*p* < 0.001). Notably, 16:0 and 18:0 were higher in aSAT compared to gSAT (*p* < 0.05). Individual MUFAs (16:1n-7, 18:1n-7, 18:1n-9, 20:1n-9) were lower in RBC-TPL compared to SAT depots (*p* < 0.001), and 16:1n-7 and 18:1n-7 were higher in gSAT than in aSAT (*p* < 0.01). In contrast, n-3 PUFAs EPA (20:5n-3), DPA n-3 (22:5n-3), DHA (22:6n-3) and total n-3 FAs were higher in RBC-TPL than in SAT depots (*p* < 0.001), with no significant difference between aSAT and gSAT (*p* > 0.05). Likewise, n-6 PUFAs DGLA (20:3n-6), AA (20:4n-6), adrenic acid (22:4n-6), DPA n-6 (22:5n-6) and total n-6 PUFA were higher in RBC-TPL than in SAT depots (*p* < 0.001), and only DGLA was higher in gSAT compared to aSAT (*p* < 0.05). Conversely, LA (18:2n-6) and eicosadienoic acid (20:2n.6) contents were lower in RBC-TPL (*p* < 0.001), with no difference between SAT depots (*p* > 0.05). Furthermore, compared to SAT depots, D5D, D6D and SCD1-18 indices were higher (*p* < 0.001), while the SCD1-16 index in RBC-TPL was lower, as mirrored by the product/precursor FA composition (*p* < 0.001). Notably, both SCD1-16 and SCD1-18 indices were higher in gSAT compared to aSAT (*p* < 0.01) with no difference for D5D and D6D indices.

### 4.3. Relationship between Fatty Acid Profiles and the VAT/SAT Ratio

**Figure 1:**
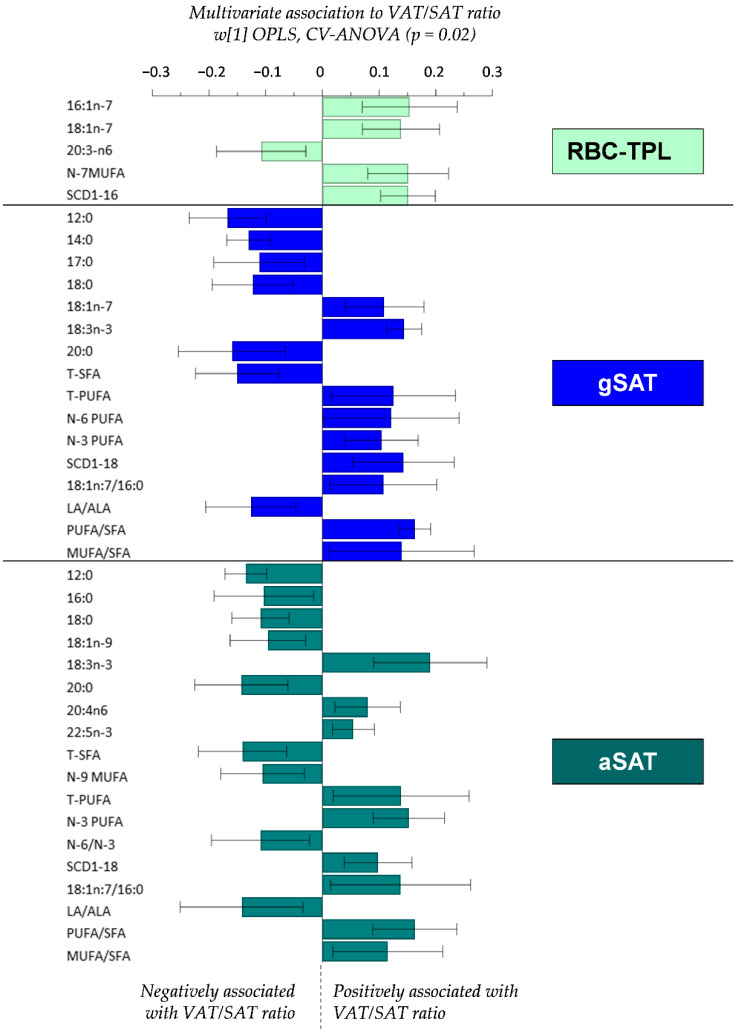
Multivariate OPLS associations between tissue-specific fatty acid composition and the VAT/SAT ratio. Only the individual and grouped FA and FA ratios in each tissue type that were significantly (95% confidence level) associated with the VAT/SAT ratio are shown in the figure. All fatty acids shown in Table 3 were included in the OPLS model. Total saturated fatty acid (T-SFA); total monounsaturated fatty acid (T-MUFA); total polyunsaturated fatty acid (T-PUFA); linoleic acid (LA); alpha-linolenic acid (ALA); stearoyl-CoA desaturase-1 index (SCD1-16: 16:1n-7/16:0; SCD1-18: 18:1n-9/18:0); 18:1n-7/16:0: vaccenic acid (elongation product of 16:1n7)/palmitic acid; gluteal subcutaneous adipose tissue (gSAT); abdominal subcutaneous adipose tissue (aSAT).

Given the differences in FA composition between RBC-TPL and SAT depots, we then explored the relationships between site-specific FA profiles and the VAT/SAT ratio (Figure 1). In the RBC-TPL, MUFAs (16:1n-7 and 18:1n-7, n-7 MUFA) and the SCD1-16 index were positively associated, while DGLA was negatively associated with the VAT/SAT ratio. The pattern of association between VAT/SAT ratio and SFA and PUFA were similar in both SAT depots. Indeed, lower SFAs (12:0, 14:0, 18:0, 20:0 and total SFA) and higher PUFAs (ALA, AA, DPA n-3, total PUFA, total n-6, total n-3) and the SCD1-18 index were associated with higher VAT/SAT ratio in both SAT depots. However, while vaccenic acid (18:1n-7) was positively associated with VAT/SAT ratio in gSAT, oleic acid (18:1n-9) and total n-9 MUFA in aSAT were inversely correlated with the VAT/SAT ratio. 

### 4.4. Relationship between Fatty Acid Profiles and S_I_

**Figure 2:**
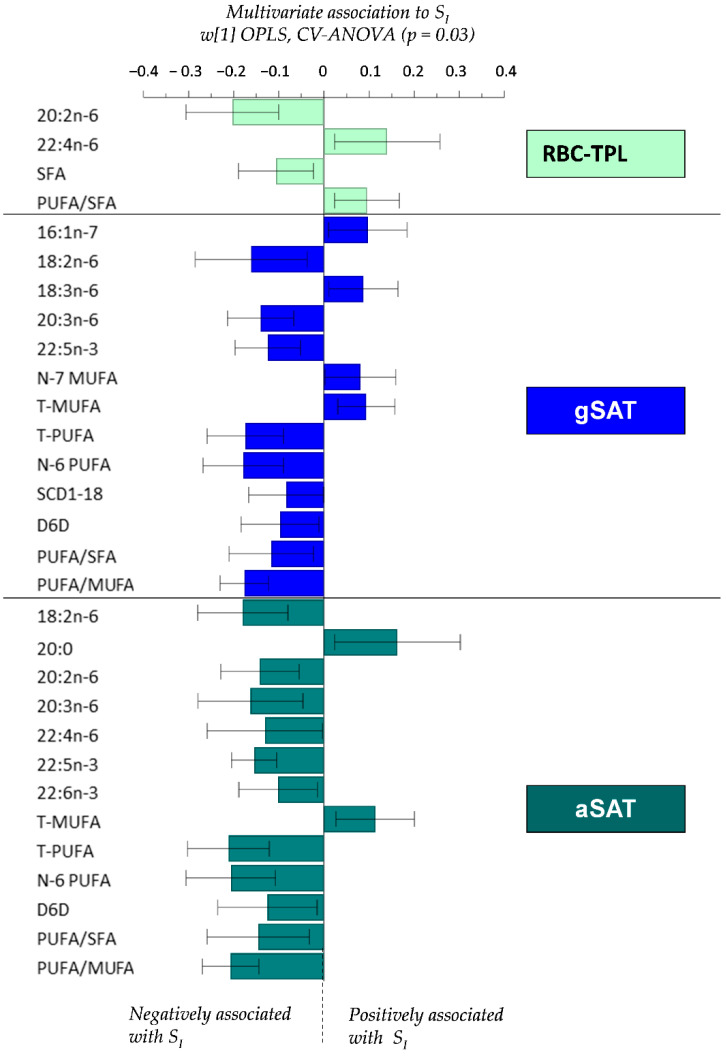
Multivariate OPLS associations between tissue-specific fatty acid composition and S_I_. Only the individual and grouped FA and FA ratios in each tissue type that were significantly (95% confidence level) associated with S_I_ are presented in the figure. All fatty acids shown in Table 3 were included in the OPLS model. Total saturated fatty acid (T-SFA); total monounsaturated fatty acid (T-MUFA); total polyunsaturated fatty acid (T-PUFA); linoleic acid (LA); alpha-linolenic acid (ALA); delta-6-desaturase index (D6D) (18:3n-6/18:2n-6); stearoyl-CoA desaturase-1 index (SCD1-16: 16:1n-7/16:0; SCD1-18: 18:1n-9/18:0); gluteal subcutaneous adipose tissue (gSAT); abdominal subcutaneous adipose tissue (aSAT).

The relationships between FA profiles (RBC-TPL and SAT depots) and S_I_ are presented in Figure 2. Higher RBC-TPL total SFA and 20:2n-6, and lower 22:4n-6 were associated with lower S_I_. In gSAT, PUFAs (LA, DGLA, DPA n-3, total n-6, total PUFA) and SCD1-18 and D6D indices were inversely correlated with S_I_, while MUFAs (16:1n-7, total n-7 and total MUFA) were positively associated with S_I_. Likewise, total MUFA in aSAT were positively associated with S_I_, while individual PUFA (LA, eicosadienoic acid, DGLA, adrenic acid, DPA n-3, DHA, total n-6, total PUFA) and the D6D index were negatively associated with S_I_.

## 5. Discussion

The key finding of this study is that the FA composition of circulating phospholipids (RBC-TPL) and SAT are distinctly associated with the centralization of body fat and S_I_ in black South African women with obesity. Notably, the FA composition of RBC-TPL was different from FA profiles in SAT. RBC-TPL contained higher proportions of SFAs and PUFAs and lower MUFAs than SAT. The higher content of SFA in RBC-TPL was not associated with the VAT/SAT ratio, but rather with lower S_I_. Furthermore, the D5D, D6D and SCD1-18 indices were higher, and the SCD1-16 index was lower in RBC-TPL compared to SAT. Interestingly, the FA composition of SAT depots differed, with higher SFAs (16:0, 18:0 and total SFA) and lower MUFAs (16:1n-7, 18:1n-7 and total MUFA), as well as lower SCD1-16 and SCD1-18 index in aSAT compared to gSAT. Despite these differences, the associations with VAT/SAT ratio and S_I_ did not differ. Indeed, in both SAT depots, lower SFAs and higher PUFAs (n-3 and n-6) correlated with a higher VAT/SAT ratio. Further, lower PUFAs (n-3 and n-6) and higher total MUFA were associated with higher S_I_. These findings confirm the hypothesis that the association of FAs with metabolic status is dependent on the FA class and the blood compartment/fraction where they occur or tissue type in which they are stored. The multivariate models were most significant for the FA composition of SAT depots, highlighting a stronger influence of SAT FA metabolism over dietary FA intake (reflected by RBC-TPL) on obesity-associated metabolic risk in these women. These findings support the contribution of excess SAT accumulation in the incidence of metabolic disorders [14,22,42]. 

The finding of high proportions of SFA and PUFA in RBC-TPL may indicate a high intake of these FA types in black SA women, which is commensurate with previous research in this population [43]. However, this was not supported by the self-reported dietary intake of the participants in this study, which reported lower SFA (9.5%E) and PUFA (8%E) intake than MUFA intake (11%E). It is important to note that under-reporting of dietary intake is well recognized, and may significantly influence investigations of nutrient patterns and association with diseases [13]. Nevertheless, SFAs such as 16:0 can be synthesized endogenously from acetyl-CoA during de novo lipogenesis, and subsequently elongated (to 18:0) and desaturated to generate MUFAs (such as 16:1 and 18:1) [14,16,38]. This pathway is elevated in individuals with obesity, and under high-carbohydrate loads and excess energy intake [14], which is characteristic of the participants in the present study. Excess consumption of carbohydrates and high glycemic load increase lipogenesis with the synthesis of SFA, especially of 16:0 and 18:0 appearing in the circulation [44,45]. Accordingly, these were the most abundant SFAs in RBC-TPL in the present study (16:0 (22%) and 18:0 (16%)). However, RBC cannot undergo de novo phospholipid synthesis or FA desaturation; these cells renew their membrane FA composition by direct exchange with plasma FFA and phospholipids pools [12]. Therefore, high content of SFA and PUFA in RBC-TPL could be derived from plasma FFAs, highly influenced by both dietary intake and endogenous synthesis in other tissues such as AT and liver. Further studies are required to elucidate the different origins of these FA classes and proportions in RBC membranes.

In contrast to higher content of SFA and PUFA in RBC-TPL, we further report lower MUFA contents in this fraction compared to SAT depots. Although, to our knowledge, no previous studies have compared the FA composition between RBC-TPL and SAT, previous data showed high SFA and PUFA and lower MUFA levels in RBC-TPL of individuals with obesity compared to lean individuals [46,47]. Moreover, we found higher indices of desaturation rates (estimated SCD1-16 activity) in SAT depots than those reflected in RBC-TPL product/precursor FA composition. SCD1-16 is involved in the endogenous synthesis of MUFA from SFA and is mainly active in AT, where it plays a central role for de novo synthesis and storage of excess energy as triglycerides [17]. Accordingly, oleic acid (18:1n-9) has been reported as the major species in adipose triglycerides [12,44,48,49,50]. Similarly, oleic acid was the most abundant FA in SAT depots (32.5%) in our study. Moreover, MUFA content in aSAT was lower (and higher SFA content) than that of gSAT in these women, which is parallel to what has been previously found in European populations [12,51]. Differences in FA metabolism between SAT depots (including synthesis, uptake, release, deposition rate, mobilisation and endogenous synthesis) have been reported [24,25,26]. These differences were attributed to their distinct FA profiles [27] and suggested to be dependent on the energy balance [51]. The higher saturation of aSAT has also been linked to the physical properties of this depot (semi-solid) concerning its anatomical role in the protection of abdominal organs [51]. Conversely, the lower content of SFA in gSAT in the present study was complemented by the higher SCD1 activity and a higher percentage of MUFA (16:1n-7, 18:1n-7 and total MUFA). This suggests a higher desaturation rate and therefore, a preferential accumulation of MUFA in gSAT. However, PUFA content was similar between aSAT and gSAT, which is supported by previous studies [12,44,51,52,53]. Long-chain PUFAs are synthesized from essential FAs (18:2n-6 and 18:3n-3) that can only be obtained from food [44,51]. These data support the notion that PUFA content in SAT depots is dependent on dietary intake, with 18:2n-6 representing the major dietary PUFA in SAT. This comparable metabolism of PUFA between aSAT and gSAT was further supported by the lack of difference in estimated desaturase activities observed in the present study.

In addition to the distinct FA profiles of RBC-TPL and SAT depots shown in the present study, it has been previously reported that the metabolic effects of individual FAs are dependent on their respective class [54,55]. For instance, while high MUFA and PUFA contents have been proposed to increase cell membrane fluidity, high contents of SFAs and low MUFAs increase membrane rigidity resulting in a reduced number of insulin receptors and binding affinity in insulin-sensitive cells [14,16]. This evidence might explain, at least in part, the different associations of FA classes with metabolic risks [16]. However, despite differential SFA and MUFA composition in SAT depots in the present study, there were no differences in the association of these FA classes with the centralization of body fat and S_I_. Indeed, lower individual and total SFAs in both SAT depots correlated with a higher VAT/SAT ratio. In contrast, higher total SFA in RBC-TPL, but not in SAT depots, was associated with lower S_I_. These findings are similar to a previous study in black SA individuals showing a positive association between SFAs in plasma phospholipids and measures of adiposity and metabolic syndrome [50]. High intake of dietary SFA may significantly increase IR via alterations of desaturases activity (mainly D5D and D9D) in cell membrane phospholipids and inhibition of insulin-stimulated Akt activation followed by the reduction in glucose uptake [16,56].

In contrast to circulating SFAs, the implication of PUFAs in the development of obesity-associated metabolic disorders is controversial. For instance, Gunes et al. showed an association between membrane enrichment in n-3 PUFA and improvement of S_I_ in overweight adolescents [14]; and plasma total n-3 PUFA was negatively associated with markers of obesity and metabolic syndrome [57,58]. Moreover, rats fed with diets rich in n-6 PUFA exhibited an increased insulin-stimulated glucose uptake in adipocytes [59]. In contrast, other studies reported positive associations between n-6 PUFA intake, obesity and metabolic syndrome [60,61], as well as inverse associations between RBC total n-6 PUFA and metabolic syndrome [62,63]. These discrepancies in the association between circulating PUFAs and metabolic status were also observed in the present study where RBC-TPL adrenic acid (22:4n-6) was positively and eicosadienoic acid (20:2n-6) was negatively correlated with S_I_. Further, both n-6 and n-3 PUFAs in SAT depots were negatively associated with S_I_ and positively correlated with the centralization of body fat (VAT/SAT ratio). Our findings are in accordance with a study in a French population showing positive associations between n-6 PUFA content of SAT (abdominal) and central fat distribution [44]. Moreover, studies in Swedish cohorts reported a positive association between individual n-3 PUFA (EPA and DHA) intake and central body fat [64] and positive correlations between aSAT n-6 PUFA (20:3n-6 and 20:4n-6) content and IR [65]. Further, Iggman et al. showed strong positive correlations between individual SFAs content in gSAT (12:0, 14:0 and 18:0) and LA with insulin sensitivity, while DHA negatively correlated with insulin sensitivity [66]. Conversely, high SFA intake was related to a higher deposition of VAT and total body fat, whereas increased dietary PUFA (mainly LA) correlated with reduced central fat accumulation [64]. These data highlight the complexity in the relationship between FA classes (especially PUFAs) and obesity-associated metabolic diseases. The role of circulating and SAT FAs species in central fat accumulation and the impairment of insulin sensitivity in different populations warrants further investigations.

The limitations of our study include the estimation of desaturase enzyme activities based on product to precursor FA ratios (indices), which may indicate a higher proportion of product than precursor and not necessarily the direct activity of these main desaturases. Moreover, the lack of detailed information on the dietary FA intake may also represent a limitation. However, the robust measure of the FA composition of RBC-TPL and two main depots of SAT represent strengths of this study. This method is regarded as more objective and accurate than estimations from dietary reporting in the investigation of the effects of these lipid molecules in the development of metabolic diseases [12,13]. This study included SA women with obesity and prospective studies in a more representative population (including men, and normal-weight individuals) with a control group are required to gain a better understanding of the associations between RBC-TPL and SAT FA composition and central fat accumulation and development of IR.

In conclusion, we show that the circulating (RBC-TPL) FA profile is distinctly different from the FA composition of SAT, with higher SFAs and PUFAs and lower MUFAs in RBC-TPL compared to the SAT depots. Notably, differences were also observed within SAT depots with higher SFAs and lower MUFAs in aSAT compared to gSAT, which were not reflected in the relationship with the VAT/SAT ratio and S_I_. SAT depots, rather than RBC-TPL, were the major contributors to the variance in central fat accumulation and insulin sensitivity. Our findings confirm the hypothesis that the FA composition of RBC-TPL and SAT is associated with metabolic risk in black SA women with obesity; and these associations are not only dependent on the FA class, but also on the tissue type and blood compartment or fraction in which they are distributed. Intervention studies are required to elucidate the causal relationship between FA composition and the metabolic profile, as well as the specific roles of individual FAs and total FA groups in mediating obesity-associated impairment of insulin sensitivity. 

## Figures and Tables

**Table 1:** Characteristics of participants.

Variables	Values (*n* = 41)
**Age (year)**	23 (21–27)
*Body composition*	
**BMI (kg/m^2^)**	33.9 ± 2.8
**Waist circumference (cm)**	103.8 ± 8.0
**Hip circumference (cm)**	116 (114–121)
**WHR**	0.9 (0.8–0.9)
**Body FM (%)**	50.4 ± 12.0
**Android FM (%)**	8.1 ± 1.1
**Gynoid FM (%)**	18.6 ± 2.0
**VAT (cm^3^)**	900 ± 345
**SAT (cm^3^)**	5454 ± 1547
**VAT/SAT**	0.2 ± 0.2
*Insulin sensitivity*	
**Fasting glucose (mmol/L)**	2777.6 (2027.6–4287.5)
**Fasting Insulin (µIU/mL)**	13.1 (8. –16.7)
**S_I_ (mU/L)^−1^min^−1^**	2.5 ± 1.4

Values are expressed as mean ± SD or median (25th–75th percentile). Body mass index (BMI); fat mass (FM); visceral adipose tissue (VAT); subcutaneous adipose tissue SAT); insulin sensitivity (S_I_).

**Table 2:** Dietary energy, macronutrient and fatty acid intake.

Variables	Median (25th–75th Percentile)
**Energy (kJ)**	12507 (9330–15374)
**Total protein (g)**	96.1 (67.5–119.9)
**Protein (%E)**	13.2 (12.2–14.1)
**Total CHO (g)**	372.7 (295.1–464.7)
**CHO (%E)**	53.1 (49.0–54.8)
**Total Fat (g)**	113.0 (71.5–131.4)
**Fat (% E)**	33.4 (30.1–36.5)
**SFA (g)**	31.4 (18.7–42.3)
**SFA (%E)**	9.5 (7.9–11.6)
**MUFA (g)**	36.0 (24.1–44.6)
**MUFA (%E)**	11.4 (10.1–12.5)
**PUFA (g)**	25.8 (18.0–32.5)
**PUFA (%E)**	7.9 (7.2–9.8)

Data presented as median (25th and 75th percentiles); *n* = 41. Kilojoules (kJ); carbohydrate (CHO); saturated fatty acid (SFA); monounsaturated fatty acid (MUFA); polyunsaturated fatty acid (PUFA).

**Table 3:** Comparison of fatty acid composition (percentage) between red blood cell total phospholipids, gluteal and abdominal subcutaneous adipose tissue depots.

	RBC-TPL	gSAT	aSAT	*p* Values
**Saturated fatty acids (SFAs)**				
14:0 (Myristic acid)	0.24 ± 0.06 ^a,b^	2.77 ± 0.62	2.89 ± 0.59	<0.001
16:0 (Palmitic acid)	21.88 ± 2.06	21.70 ± 1.12	22.52 ± 1.11 ^c^	0.024
18:0 (Stearic acid)	16.31 ± 0.78 ^a,b^	5.25 ± 1.53	5.75 ± 1.49 ^c^	<0.001
20:0 (Arachidic acid)	0.35 ± 0.04 ^a,b^	0.16 ± 0.06	0.16 ± 0.05	<0.001
22:0 (Behenic acid)	1.39 ± 0.20 ^a,b^	0.06 ±0.03	0.05 ± 0.01	<0.001
24:0 (Lignoceric acid)	3.99 ± 0.64 ^a,b^	0.05 ± 0.02	0.04 ± 0.01	<0.001
Total SFAs	44.16 ± 1.68 ^a,b^	30.76 ± 2.88	32.23 ± 2.46 ^c^	<0.001
**Mono-unsaturated fatty acids (MUFAs)**				
16:1n-7 (Palmitoleic acid)	0.24 ± 0.07 ^a,b^	6.61 ± 2.09 ^c^	5.93 ± 1.68	<0.001
18:1n-7 (cis-Vaccenic acid)	1.11 ± 0.22 ^a,b^	3.00 ± 0.49 ^c^	2.79 ± 0.43	<0.001
18:1n-9 (Oleic acid)	10.70 ± 0.87 ^a,b^	32.56 ± 0.92	32.49 ± 1.01	<0.001
20:1n-9 (Eicosenoic acid)	0.18 ± 0.02 ^a,b^	0.71 ± 0.10	0.69 ± 0.11	<0.001
Total n-7 MUFAs	1.34 ± 0.27 ^a,b^	9.61 ± 2.50 ^c^	8.71 ± 2.02	<0.001
Total n-9 MUFAs	14.23 ± 0.89 ^a,b^	33.31 ± 0.94	33.22 ± 1.04	<0.001
Total MUFAs	15.57 ± 0.97 ^a,b^	42.92 ± 2.64 ^c^	41.93 ± 2.25	<0.001
**Poly-unsaturated fatty acids (PUFAs)**				
20:5n-3 (EPA)	0.47 ± 0.23 ^a,b^	0.09 ± 0.04	0.08 ± 0.04	<0.001
22:5n-3 (DPA n-3)	1.96 ± 0.31 ^a,b^	0.27 ± 0.09	0.24 ± 0.08<	<0.001
22:6n-3 (DHA)	4.45 ± 0.81 ^a,b^	0.24 ± 0.08	0.21 ± 0.08<	<0.001
18:2n-6 (LA)	11.71 ± 1.31 ^a,b^	22.33 ± 1.64	22.15 ± 1.55	<0.001
20:2n-6 (Eicosadienoic acid)	0.35 ± 0.04 ^a,b^	0.50 ± 0.10	0.48 ± 0.12	<0.001
20:3n-6 (DGLA)	1.53 ± 0.22 ^a,b^	0.43 ±0.12 ^c^	0.37 ± 0.12	<0.001
20:4n-6 (AA)	15.74 ± 1.19 ^a,b^	0.73 ±0.18	0.67 ± 0.17	<0.001
22:4n-6 (Adrenic acid)	3.37 ± 0.59 ^a,b^	0.30 ± 0.09	0.27± 0.09	<0.001
22:5n-6 (DPA-n-6)	0.68 ± 0.16 ^a,b^	0.08 ± 0.04	0.08 ± 0.03	<0.001
Total n-3	6.90 ± 1.87 ^a,b^	1.76 ± 0.33	1.66 ± 0.32	<0.001
Total n-6	33.38 ± 1.87 ^a,b^	24.56 ± 1.73	24.18 ± 1.64	<0.001
Total PUFAs	40.27 ± 1.77 ^a,b^	26.32 ± 1.95	25.84 ± 1.80	<0.001
**Estimated enzyme activities/indices**				
D5D	10.57 ± 1.95 ^a,b^	1.79 ± 0.43	1.90 ± 0.37	<0.001
D6D	0.13 ± 0.02 ^a,b^	0.019 ± 0.01	0.016 ± 0.01	<0.001
SCD1-16	0.01 ± 0.00 ^a,b^	0.31 ± 0.11 ^c^	0.27 ± 0.08	<0.001
SCD1-18	0.68 ± 0.01 ^a,b^	0.65 ± 0.30 ^c^	0.53 ± 0.21	<0.001

Fatty acids (FA) presented as relative percentages of total FAs (%, wt:wt) and mean ± SD (*n* = 41). *p*-Values represent the differences in FA composition between the sites using one-way repeated measures ANOVA as follows: ^a^: RBC vs. gSAT; ^b^: RBC vs. aSAT and ^c^: gSAT vs. aSAT. Linoleic acid (LA); eicosapentaenoic acid (EPA); docosapentaenoic acid (DPA); docosahexaenoic acid (DHA); dihomo-gamma-linolenic acid (DGLA); arachidonic acid (AA); saturated fatty acid (SFA); monounsaturated fatty acid (MUFA); polyunsaturated fatty acid (PUFA); delta-5-desaturase index (D5D) (20:4n-6/20:3n-6); delta-6-desaturase index (D6D) (18:3n-6/18:2n-6); stearoyl-CoA desaturase-1 (SCD1-16: 16:1n-7/16:0; SCD1-18: 18:1n-9/18:0).
